# When do defecation function and quality of life recover for patients with non-ostomy and ostomy surgery of rectal cancer?

**DOI:** 10.1186/s12893-020-00719-6

**Published:** 2020-03-30

**Authors:** Guojun Tong, Guiyang Zhang, Jian Liu, Zhaozheng Zheng, Yan Chen, Min Li, Yan Zhong, Pingping Niu, Xuting Xu

**Affiliations:** 1 Department of Colorectal Surgery, Huzhou Central Hospital, Zhejiang, 313000 China; 2 Central Laboratory, Huzhou Central Hospital, Zhejiang, 313000 China; 3 Vice President of Huzhou Central Hospital, Zhejiang, 313000 China; 4 Huzhou Central Hospital, Zhejiang, 313000 China

**Keywords:** Rectal cancer, QOL, Defecation function

## Abstract

**Background:**

Rectal cancer (RC) surgery often results in permanent colostomy, seriously limiting the quality of life (QOL) in patients in terms of bowel function. This study aimed to examine defecation function and QOL in RC patients who underwent non-ostomy or ostomy surgery, at different time-points after surgery.

**Methods:**

In total, 82 patients who underwent an ostomy and 141 who did not undergo an ostomy for the treatment of RC at our colorectal surgery department between January 2013 and January 2015 were enrolled. Surgical methods, tumor distance from the anal margin (TD), anastomosis distance from the anal margin (AD) and complications were compered between the non-ostomy and ostomy surgery groups. QOL was compared between the two groups at years 2, 3, and 4 after surgery. The Wexner score and the validated cancer-specific European Organization for Research and Treatment of Cancer (EORTC QLQ-CR30) questionnaire scores were assessed for all patients in January 2017. SPSS 21.0 was utilized for all data analyses.

**Results:**

Surgical methods, TD, and AD significantly differed between the non-ostomy and ostomy surgery groups (all *P* < .001). However, no differences were found in the number of complications between the groups (*P* = .483). For the 192 patients undergoing Dixon surgery, role function (RF), global QOL (GQOL), sleep disturbance, and the incidence of constipation showed significant differences between the two groups (*P* = .012, *P* = .025, *P* = .036, and *P* = .015, respectively). In the 31 cases of permanent ostomy, we observed significant differences in GQOL scores, dyspnea incidence, and financial difficulties across the different years (*P* = .002, *P* = .036, and *P* < .01, respectively). Across all 223 cases, there were significant differences in social function and GQOL scores in the second year after surgery (*P* = .014 and *P* < .001, respectively). However, no differences were observed in the other indices across the three time-points.

**Conclusions:**

RC patients undergoing ostomy surgery, especially those with low and super-low RC, revealed poorer defecation function and QOL in the present study. However, 2 years after surgery, most of the defecation and QOL indicators showed recovery.

## Background

Rectal cancer (RC) surgery often results in permanent colostomy, seriously limiting the quality of life (QOL) of patients, in terms of bowel and sexual function [1, 2]. With advances in RC surgery and the medical equipment used, the efficacy of low and ultra-low anal surgery is being recognized by an increasing number of surgeons and patients. Therefore, there has been an increase in the use of preventive ostomy surgery following rectal surgery. However, this has led to concerns pertaining to defecation function and QOL. Yang et al. [3] demonstrated that the first postoperative month was crucial for patient recovery, and that the QOL had not yet fully recovered 6 months after permanent colostomy. A vast body of literature suggests that patients with colostomy have a worse QOL than those without an ostomy [4–14]. The differences in outcome between those who undergo an ostomy surgery (including preventive and permanent ostomy surgery) and those who do not, in terms of defecation function and QOL are still uncertain.

The validated cancer-specific European Organization for Research and Treatment of Cancer (EORTC QLQ-CR30) questionnaire is useful in the evaluation of curatively treated patients with RC [15]. In this study, we used the Wexner score [16] and QLQ-CR30 to evaluate differences in defecation function and QOL between those who underwent an ostomy and those who did not for RC across different time-spans in order to explore when the defecation and QOL can restore.

## Methods

### Patients

**Fig. 1:**
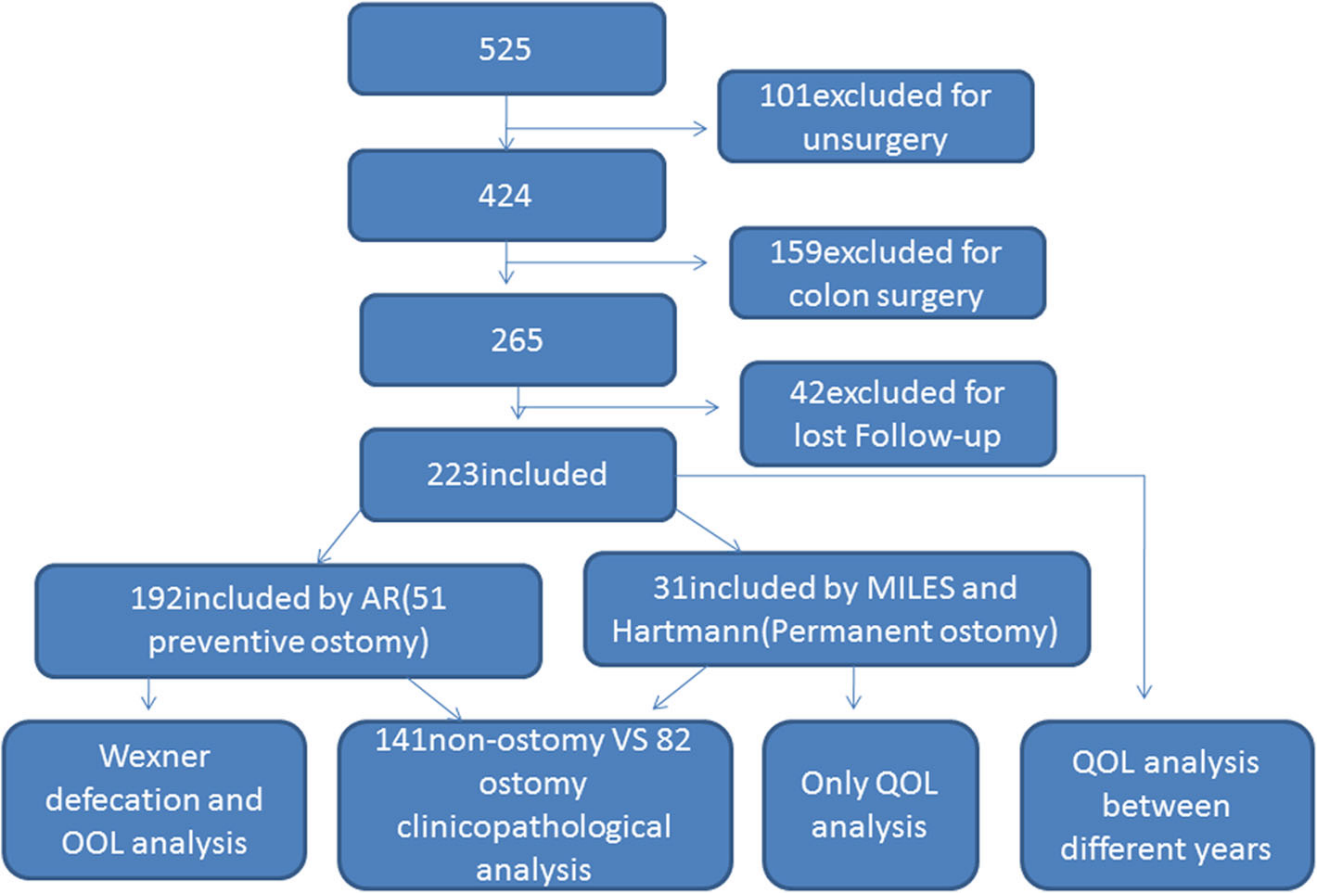
Flow chart and study methodology of recruitment and participation in this study

We collected data on 525 colorectal cancer patients from January 2013 to January 2015 from the colorectal surgery department of our hospital. We excluded data for 101 cases with un-surgery and for 159 cases that required colon cancer surgery. Furthermore, we excluded data for 42 patients who were lost to follow-up. A total of 223 cases were finally included; 192 patients had undergone anterior resection with anastomosis (AR) surgery including 51 cases involving preventive ostomy and 31 cases involving permanent ostomy surgery (MILES and Hartmann operation). Preventive ostomy patients were all closed within 1 year based on our data. The inclusion criteria were as follows: patients who had undergone RC radical surgery, presence of complete follow-up data, and active cooperation from patients or their family members. Exclusion criteria were as follows: patient death, incomplete clinicopathological and follow-up data, lack of cooperation from patients or their family members (Fig. 1). A total of 141 cases were assigned to the non-ostomy surgery group and 82 cases to the ostomy surgery (51 preventive ostomy and 31 permanent ostomy) group.

### Follow-up and clinical data

Clinicopathological data included details on sex, age, preoperative C-reactive protein (CRP) level, maximum tumor diameter, bowel resection length, use of laparoscopic surgery, surgical method, tumor distance from the anal margin (TD), anastomosis distance from the anal margin (AD), TNM staging, tumor differentiation, and chemotherapy. Clinicopathological features and complications were compared between the ostomy and non-ostomy surgery groups. Clinical data were recorded using Haitai doctor work software V3.0 (Nanjing Haitai Information Medical Co., Ltd.).

The Wexner and EORTC QLQ-CR30 questionnaire are well-established tools to assess the QOL of cancer patients and their reliability and validity have been proven [17, 18]. Hence these were administered to the patients and all responses were entered by us. Telephone calls or emails or both were the predominant methods used for follow-up. The questionnaires were filled by a physician rather than the patients themselves, to avoid errors owing to potential misunderstandings by the patients. The defecation function and QOL were compared between the ostomy and non-ostomy surgery groups among the 192 patients that underwent AR surgery. Only QOL was compared across years 2,3,4 after surgery among the 31 permanent ostomy patients. Furthermore, for all patients, the QOL was compared between the non-ostomy and ostomy surgery groups at years 2, 3 and 4 after surgery (See Fig. 1).

In order to allow for the comparison of the scores in each field, linear transformation was further performed using the extreme difference method, and the crude score was converted into a standard score (SS) within a 0–100 range. Transformation was also employed to change the direction of the score. In the QLQ-CR30 scale, with the exception of items 29 and 30, reverse entry was used (larger the value, worse the QOL), as clearly stated in the scoring rules: the higher the score for the functional field and the overall health field, the better the functional status and QOL, and higher scores in the symptom area indicate the presence of a larger number of symptoms or problems (worse QOL). Therefore, the standardization of functional areas of calculations also needed to change direction. Specifically, they were calculated using the following formula (where ‘R’ is the total distance between each field or item):

Functional area: SS = [1-(RS-1)/R] × 100; Symptom area and general health area: SS = [(RS-1)/R] × 100. The QOL score was standardized according to the SS.

Functional area included Physical function (PF), Role function (RF), Emotional function (EF), Cognitive function (CF), Social function (SF), Global QOL; symptom area and health area included fatigue, nausea and vomiting, pain, dyspnea, sleep disturbance, appetite loss, constipation, diarrhea, and financial difficulties.

### Follow-up

The retrospective study included assessments that were performed at one point across three different timespans: 2, 3, and 4 years after surgery. Follow up was performed on January 2017. All participants were informed of the goals of the study and method of data collection via telephonic or email communication or both and were invited to participate. All patients were assured that refusal to participate would not prevent the provision of treatment at the hospital.

### Statistical analysis

All data on clinicopathology, the Wexner score and QOL were coded and entered into SPSS 21.0 for windows (IL, USA). Continuous data were tested using an independent sample t-test between the two groups, and analysis of variance and an F test were used to compare more than two groups. The mean and standard deviation (SD) are expressed as^−^x ± s. Count data were dealt with using CROSSTAB and a chi-square test. A line chart was used for measurement data analysis. When the observed number of count data was less than five, Fisher’s exact test was utilized. *P* < .05 was accepted as the level of significance.

## Results

### Comparison of the clinicopathological data between the ostomy and non-ostomy surgery groups

The total population of 223 patients comprised 145 men (65%) and 75 women (35%), with an average age of 63.84 ± 9.46 years. Of these 82 patients underwent ostomy and 141 patients underwent non-ostomy surgery. No statistical differences were noted between the ostomy and non-ostomy surgery groups in terms of sex, age, preoperative CRP level, maximum tumor diameter, bowel resection length, laparoscopic surgery, and chemotherapy (*P* = .192, *P* = .286, *P* = .100, *P* = .903, *P* = .873, *P* = .100, *P* = .192, respectively). Fisher’s exact test revealed no statistical differences in the TNM staging and tumor differentiation between the two groups (*P* = .259, *P* = .477). In terms of the surgical method used, the tumor distance from the anal margin (TD) and anastomosis distance from the anal margin (AD) differed significantly between the two groups (*P* < .001, *P* < .001, *P* < .001). The number and percentage of counting data and the mean and standard deviation of the clinicopathological data were evaluated in both groups. Further details are shown in Table 1.

**Table 1:** Comparison of clinical and pathological indices of rectal cancer in ostomy group and non – ostomy group^−^x ± s (%)

Factor	Ostomy group(82)	Non-ostomy group(141)	P
Gender			0.192
Male	58(40.0)	87(60.0)	
Female	24(30.8)	54(69.2)	
Age	64.73 ± 9.05	63.33 ± 9.69	0.286
Preoperative CRP	2.83 ± 3.10	5.81 ± 20.95	0.100
Tumor max diameter	4.60 ± 2.09	4.57 ± 1.80	0.903
Bowel resection length	21.20 ± 2.59	21.13 ± 3.69	0.873
Laparoscopic surgery			0.100
No	62(40.5)	91(59.5)	
Yes	20(28.6)	50(71.4)	
Operation method			< 0.001 ^a^
AR	51(26.6)	141(73.4)	
MILES	27(100.0)	0(0.0)	
Hartmann	4(100.0)	0(0.0)	
TD^a^	5.39 ± 2.99	11.49 ± 4.22	< 0.001**
AD^b^	2.04 ± 2.25	6.52 ± 3.75	< 0.001**
TNM staging			0.259^a^
0	2(66.7)	1(33.3)	
I	25(41.7)	35(58.3)	
II	32(37.2)	54(62.8)	
III	22(30.1)	51(69.9)	
IV	1(100.0)	0(0.0)	
Tumor differentiation			0.477^a^
Well	2(50.0)	2(50.0)	
Moderate	67(35.1)	124(64.9)	
Moderate-low	5(38.5)	8(61.5)	
Low or non	8(53.3)	7(46.7)	
Chemotherapy			0.192
Yes	64(35.2)	118(64.8)	
No	18(43.9)	23(56.1)	

^*^Fisher exact test, **P < 0.05Statistically significant

^a^:Tumor distance from the anal margin

^b^:Anastomosis distance from the anal margin

### Comparison of the complications between the ostomy and non-ostomy surgery groups

Table 2 reveals the rate of preoperative complications in the two groups. A total of seven cases (8.54%) in the ostomy group and 12 (8.51%) in the non-ostomy group presented such complications; the difference between the two groups was not significant (*P* = 0.483).

**Table 2:** Comparisons of complications of rectal cancer in ostomy group and non – ostomy group

complications	Ostomy group (*n* = 82)	Non-ostomy group (*n* = 141)	P
Total(%)	7(8.54)	12(8.51)	0.483
Anastomotic leakage	0	4	
Anastomotic stenosis	2	0	
Urinary tract injury or infection	2	4	
Intestinal obstruction	2	3	
Lung infection	1	1	

### Comparison of defecation function and QOL in the ostomy and non-ostomy groups among AR surgery patients

**Fig. 2:**
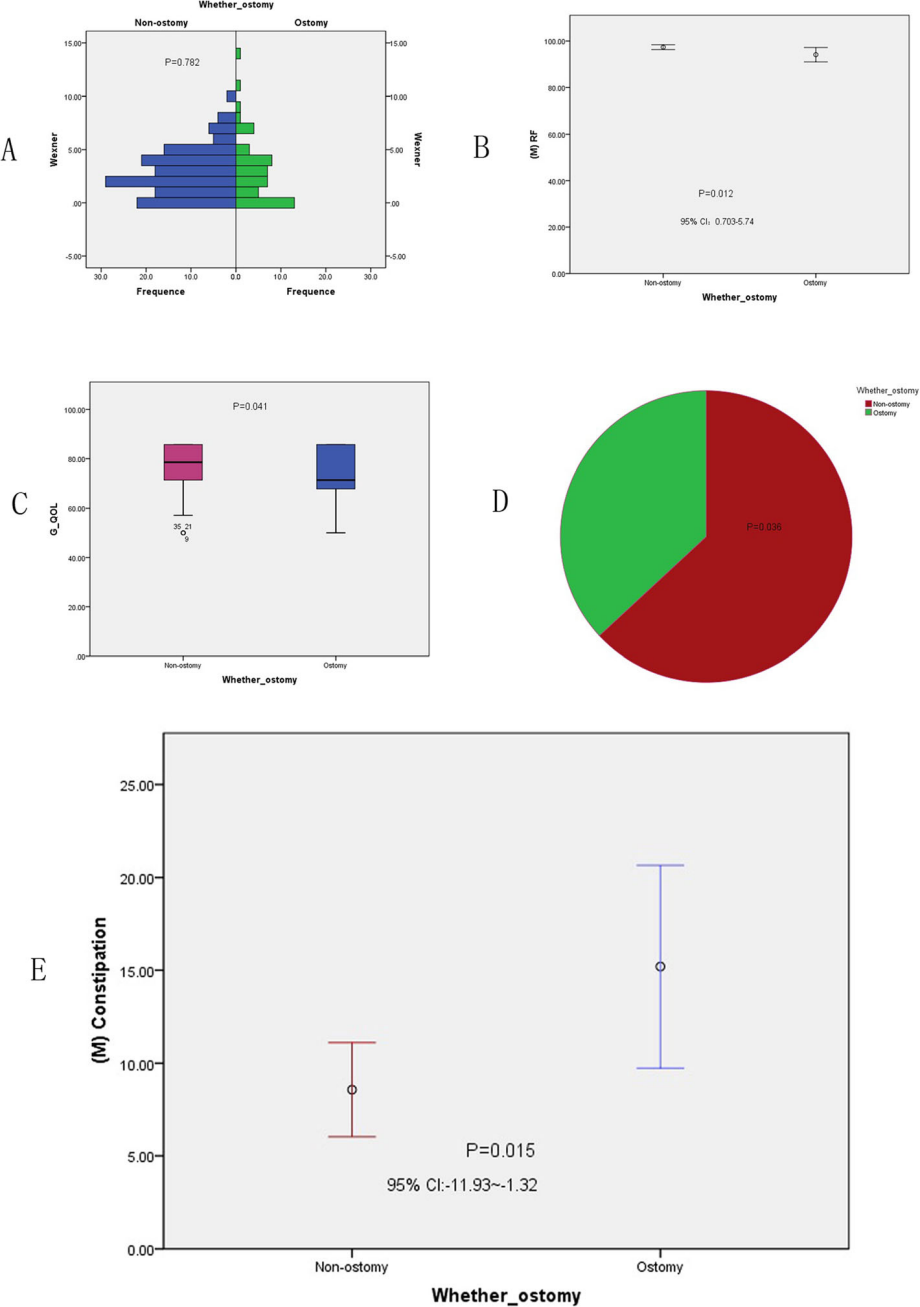
Comparison of Wexner score,Functional area and Symptom area between non-ostomy group and ostomy group for 192 AR patients. **a**: About Wexner score,there were no significant difference between non-ostomy and ostomy group, F = .077,*P* = .782. **b**: RF was significantly different (*P* = .012), **c**: The total health area (G-QOL) was significantly different between the two groups, *P* = .025. **d**: Sleep disturbance showed significant difference,*P* = .036. **e**: Constipation showed significant difference, *P* = .015

In the present study, 192 patients (86.1%) with AR surgery were enrolled, comprising 141 non-ostomy and 51 ostomy patients. Their Wexner score (Fig. 2 a) and QOL were compared. We observed significant differences between the two groups in terms of role function (RF) (Fig. 2 b), global QOL (GQOL) (Fig. 2 c), sleep disturbance (Fig. 2 d), and constipation (Fig. 2 e) (*P* = .012, *P* = .041, *P* = .036, *P* = .015, respectively); no significant differences were noted for the other indices (see Table 3) .

**Table 3:** Comparison of 192 AR patients between non-ostomy rectal cancer and ostomy rectal cancer, Mean (SD)

EORTC QLQ-CR30 and Wexner Score	Non-ostomy (*N* = 141)	Ostomy (*N* = 51)	P
Wexner	2.96(2.27)	3.08(3.08)	0.782
PF	98.03(4.58)	97.06(4.58)	0.212
RF	97.34(6.30)	94.11(11.00)	0.012*
EF	94.90(8.14)	93.06(8.78)	0.177
CF	97.03(7.11)	96.08(7.28)	0.417
SF	94.15(10.62)	90.93(13.48)	0.087
G_QOL	77.76(9.43)	74.09(11.25)	0.041*
Fatigue	7.50(8.38)	6.50(8.29)	0.317
Nausea_Vomiting	1.77(6.94)	2.20(6.94)	0.703
Pain	3.28(7.26)	3.92(8.10)	0.601
Dyspnea	4.96(11.27)	7.35(12.54)	0.210
Sleep_disturbance	7.27(12.15)	11.76(15.29)	0.036*
Appetite_loss	4.08(10.62)	5.39(10.38)	0.447
Constipation	8.57(15.22)	15.19(19.42)	0.015*
Diarrhea	2.14(7.64)	4.41(9.63)	0.093
Financial_difficulties	10.18(16.12)	15.68(19.98)	0.052

*PF* Physical function, *RF* Role function, *EF* Emotional function, *CF* Cognitive function, *SF* Social function, *GQOL* globe quality of life;*P < 0.05Statistical differences

### Analysis of QOL across the different years for permanent ostomy

**Fig. 3:**
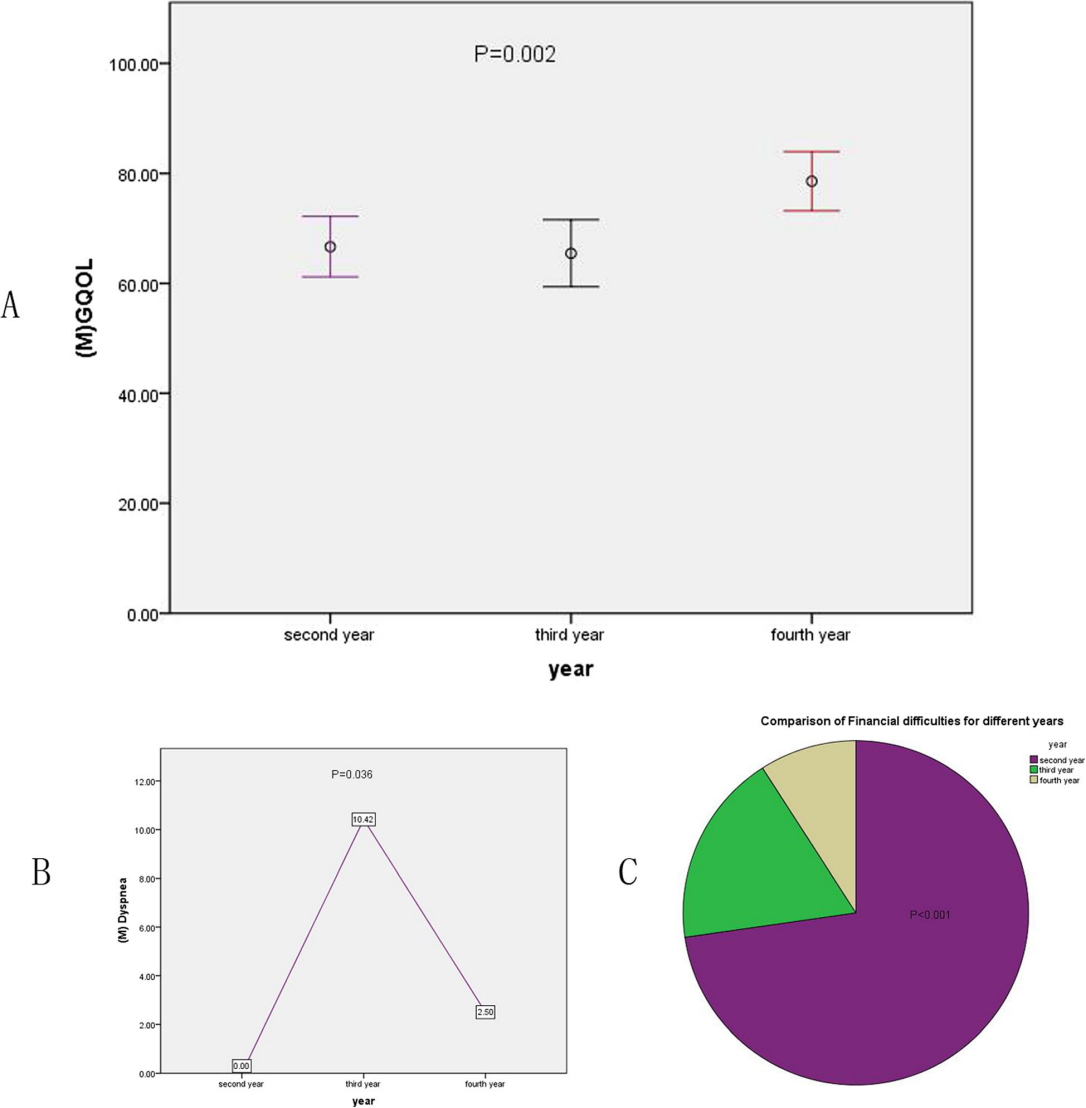
Comparison of Functional area and Symptom area between different year span for 31 permanent ostomy patients. Most indices showed no significance except for GQOL (**a**), Dyspnea (**b**) and Financial difficulties (**c**) (*P* = .002,*P* = .036,*P* < .001)

A total of 31 (13.9%) patients with permanent fistula (MILES and Hartmann) were analyzed, and their QOL was assessed over the different years. The mean value, SD, and *P* value were evaluated. Significant differences were noted for GQOL (Fig. 3 a), dyspnea (Fig. 3 b), and financial difficulties (Fig. 3 c) (*P* = .002, *P* = .036, *P* < .001); no significant differences were noted for the other indices (details were shown in Table 4).

**Table 4:** Comparison of QOL in different years for 31 Hartman and MILES patients, Mean (SD)

EORTC QLQ-CR30	Second year (*n* = 9)	Third year (*n* = 12)	Fourth year (*n* = 10)	P
PF	96.67(8.29)	97.50(5.00)	99.50(1.58)	0.514
RF	95.83(8.34)	96.88(7.77)	98.75(3.95)	0.666
EF	84.03(32.19)	91.67(8.57)	98.13(4.22)	0.258
CF	97.22(8.33)	96.87(5.65)	100(0.00)	0.404
SF	75.00(10.83)	83.33(15.39)	90.00(16.45)	0.100
G_QOL	66.67(7.14)	65.47(7.14)	78.57(7.53)	0.002*
Fatigue	8.31(12.48)	9.73(10.56)	7.53(8.32)	0.883
Nausea_Vomiting	2.78(5.51)	1.04(3.61)	0.00(0.00)	0.277
Pain	4.167(8.84)	5.21(8.36)	5.00(6.45)	0.954
Dyspnea	0.00(0.00)	10.41(12.87)	2.50(7.91)	0.036*
Sleep_disturbance	5.56(11.02)	12.50(22.61)	7.50(12.07)	0.619
Appetite_loss	5.56(11.02)	6.25(11.31)	0.00(0.00)	0.257
Constipation	2.78(8.33)	4.17(9.73)	0.00(0.00)	0.441
Diarrhea	0.00(0.00)	4.12(9.73)	2.50(7.91)	0.883
Financial_difficulties	22.22(8.33)	4.17(9.73)	2.50(7.91)	< 0.001*

### Analysis of QOL among all patients in the ostomy and non-ostomy groups across the different years

**Fig. 4:**
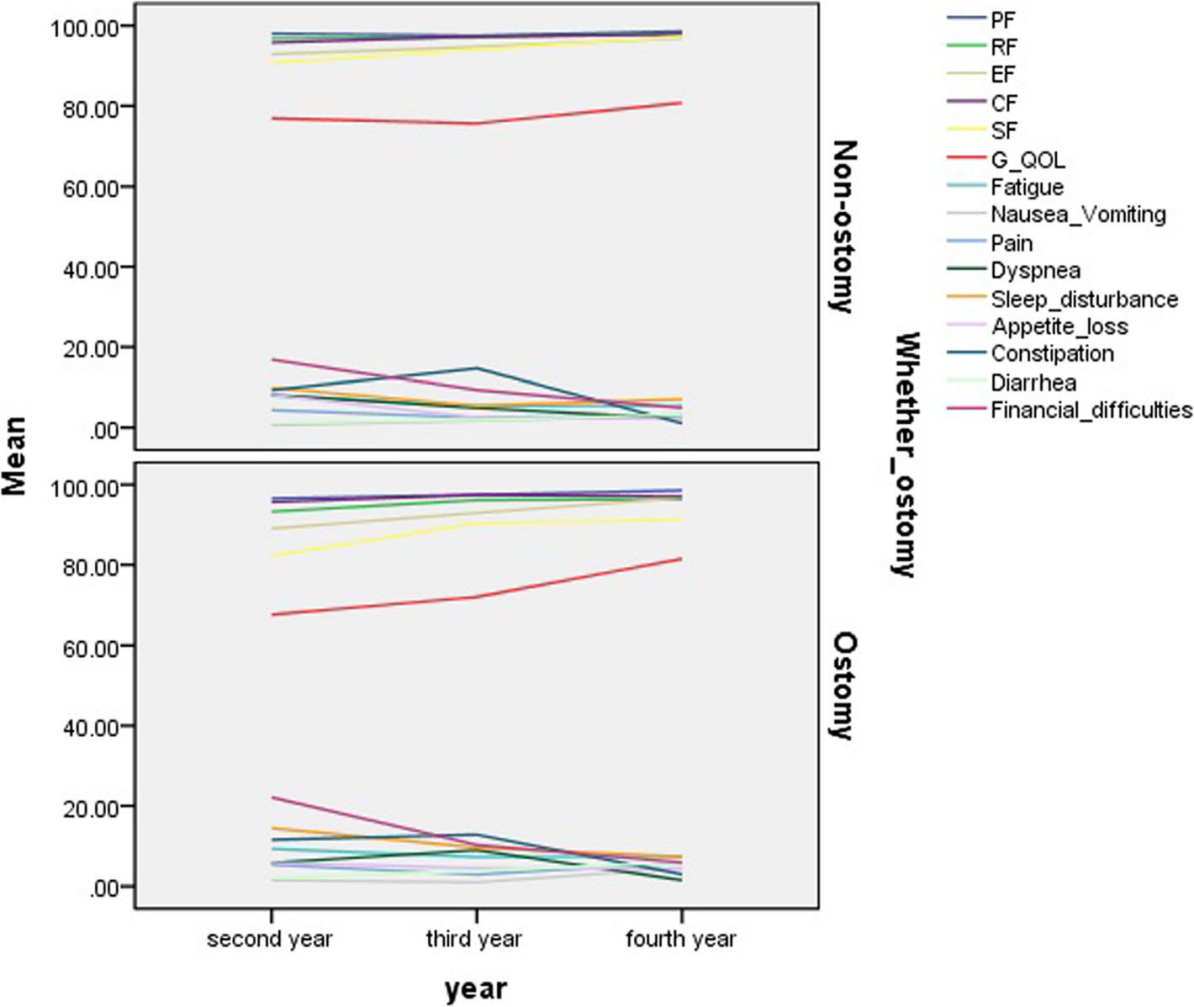
Function and symptom indices line charts for total patients in three different time span. Comparison between non-ostomy and ostomy groups were carried out too .In the second year after operation, SF and GQOL were significantly different (*P* = .014, *P* < .001), and there were no differences in other functional and symptom areas (all *P* > .05); At postoperative 3rd and 4th years all indicators in the functional and symptom areas were not statistically significant (all P > .05). See Table 5 for details

There were 70 cases (44 cases in the non-ostomy group, 26 in the ostomy group) in the second year, 90 cases (51 cases in the non-ostomy group, 39 in the ostomy group) in the third year, and 63 cases (46 cases in the non-ostomy group, 17 in the ostomy group) in the fourth year. The mean values of each indicator of the QLQ-CR30 between the two groups across the different time periods are presented in a line graph (Fig. 4). Table 5 shows the mean value, SD, and *P* value of each indicator of the QLQ-CR30 for each time-point.

**Table 5:** Comparison of QOL indices in different years for total rectal cancer in ostomy group and non-ostomy group^−^x ± s

EORTC QLQ-CR30 (*n* = 223)	Second year(70)			Third year(90)			Fourth year(63)		
	Non-ostomy (44)	ostomy(26)	P	Non-ostomy (51)	Ostomy group (39)	P	Non-ostomy (46)	Ostomy(17)	P
PF	98.07 ± 4.73	96.53 ± 6.28	0.252	97.5 ± 6.42	97.44 ± 5.11	0.953	98.59 ± 3.90	98.53 ± 3.86	0.959
RF	96.88 ± 6.67	93.27 ± 11.85	0.163	97.50 ± 6.42	96.15 ± 9.14	0.583	98.10 ± 5.87	96.32 ± 7.35	0.325
EF	93.04 ± 8.86	89.04 ± 19.94	0.252	94.73 ± 7.84	92.95 ± 8.62	0.309	96.88 ± 7.42	96.69 ± 6.67	0.929
CF	95.88 ± 8.83	95.67 ± 8.62	0.924	97.30 ± 6.28	97.44 ± 5.11	0.915	97.83 ± 6.07	97.28 ± 7.87	0.671
SF	90.91 ± 13.31	82.21 ± 15.07	0.014*	94.12 ± 9.47	90.38 ± 14.18	0.139	97.28 ± 7.87	91.18 ± 13.08	0.085
GQOL	77.11 ± 9.06	67.58 ± 8.63	< 0.001*	75.63 ± 9.28	71.98 ± 11.52	0.099	80.75 ± 9.39	81.51 ± 6.71	0.759
Fatigue	7.75 ± 8.70	9.27 ± 9.53	0.498	5.39 ± 7.97	7.26 ± 9.21	0.306	5.42 ± 8.10	7.35 ± 8.29	0.407
Nausea_Vomiting	0.57 ± 3.77	1.44 ± 4.07	0.366	1.72 ± 6.62	0.96 ± 4.43	0.514	2.99 ± 9.20	4.41 ± 9.82	0.595
Pain	4.26 ± 8.50	5.29 ± 8.78	0.631	2.45 ± 6.62	2.88 ± 6.70	0.760	3.26 ± 6.68	5.88 ± 8.97	0.284
Dyspnea	7.95 ± 12.95	5.77 ± 10.74	0.471	4.90 ± 12.27	8.97 ± 13.44	0.143	2.17 ± 7.12	1.47 ± 6.06	0.719
Sleep_disturbance	9.66 ± 12.31	14.42 ± 17.57	0.188	5.39 ± 11.53	9.39 ± 11.53	0.164	7.07 ± 12.54	7.35 ± 11.74	0.935
Appetite_loss	7.95 ± 10.95	5.77 ± 17.57	0.188	5.39 ± 11.53	9.39 ± 11.53	0.164	2.17 ± 8.86	4.41 ± 9.82	0.391
Constipation	9.30 ± 13.39	11.54 ± 17.65	0.554	14.71 ± 19.48	12.82 ± 18.91	0.646	1.09 ± 5.15	2.94 ± 8.30	0.399
Diarrhea	1.16 ± 5.33	1.92 ± 0.79	0.607	1.96 ± 6.79	3.85 ± 9.14	0.264	3.26 ± 10.01	5.88 ± 10.93	0.372
Financial_difficulties	16.86 ± 17.01	22.12 ± 11.71	0.181	9.31 ± 16.55	10.26 ± 19.63	0.806	4.89 ± 12.49	5.88 ± 10.93	0.774

In the second year, function scores such as the social function (SF) and GQOL scores differed significantly between the non-ostomy and ostomy groups (*P* = .014 and < .001), and the mean SF and GQOL scores in the non-ostomy group were higher than those in the ostomy group; no significant differences were observed in the other function scores such as the PF, RF, EF, and cognitive CF scores. There were no statistical differences in the symptom scores, such as those pertaining to fatigue, nausea and vomiting, pain, dyspnea, sleep disturbance, appetite loss, constipation, diarrhea, and financial difficulties, between the two groups (*P* = .498, *P* = .366, *P* = .631, *P* = .471, *P* = .188, *P* = .188, *P* = .554, *P* = .607, *P* = .181, respectively). There were no significant differences between the two groups in terms of the function and symptom scores in the third and fourth years (all *P* > .05).

## Discussion

The goal of the present study was to retrospectively compare defecation function and QOL between RC patients undergoing an ostomy and those not undergoing an ostomy, 2, 3, and 4 years after surgery, using the Wexner score and EORTC QLQ-CR30 questionnaire.

With the increasing use of low and ultra-low anorectal surgery, the rates of preventive ostomy have also risen. These medical procedures result in changes that affect all aspects of patients’ lives. Beaubrun En Famille Diant et al. highlighted the importance of physical self-esteem in temporary ostomy and the role of good body image and substantial emotional self-esteem in permanent ostomy [19]. For patients undergoing closed operation and permanent fistula procedures, defecation function and QOL are matters of great concern. Thus, we aimed to examine when defecation function and QoL recovers in such patients. Camilleri-Bernnan et al. [20] demonstrated that gastrointestinal symptoms in stoma patients begin to show improvement in the third postoperative month; however, they did not demonstrate when defecation function and QOL would be restored among patients undergoing preventive and permanent ostomy. Schmidt [21] also showed that the QOL scores in the early postoperative period were much lower than those at baseline. He illustrated that patients’ global health, QOL, EF, and physical function begin to improve in the third month, and that it takes 2 years for the nausea and vomiting, agrypnia, constipation, and diarrhea scores to revert to baseline values. Reyes et al. [22] found significant differences in health-related quality of life (HR-QOL) among racial groups, with African-Americans showing the worst HR-QOL, which was measured using physical and mental composite scores.

Our study demonstrated that there was no significant difference in sex, age, preoperative CRP levels, maximum tumor diameter, bowel resection length, TNM stage, tumor differentiation, and chemotherapy between the two groups, although surgical method, TD, and AD differed. This may be related to the complete preservation of the rectal sphincter during surgery. These data suggest that preventive ostomy was mainly used in cases of low and ultra-low RC. Several studies have reported the importance of preventive ostomy in low or super-low rectal surgery [23–27]. For RC patients with diabetes, those who have undergone neoadjuvant chemoradiotherapy, and those with a distance between the tumor and anal edge ≤5 cm, anastomotic leakage after the anterior resection of RC must be paid attention to. When necessary, preventive colostomy or the use of a double-perfusion cannula for abdominal flushing should be considered [28].

Complications following low RC surgery are a matter of concern. Awareness on anastomotic complications after intersphincteric resection should be increased, especially among male patients with radiation colitis [29]. In our study, we analyzed the presentation of postoperative complications and found that there were seven cases of complications (8.54%) in the ostomy group, including two cases of anastomosis stenosis, two of urinary system injury or infection, two of postoperative intestinal obstruction, and one of pulmonary infection. However, there were no cases of anastomosis leakage. In the non-ostomy group, there were 12 cases of complications (8.51%), including four of anastomosis leakage, four of urinary system injury or infection, three of postoperative intestinal obstruction, and one of pulmonary infection. However, there were no cases of anastomosis stenosis. Hence, the number of anastomosis leakage cases decreased to 0 in the ostomy group, although the number of anastomosis stenosis cases increased to 2. We also noted that the number of total complications in the ostomy group was lower than that in the non-ostomy group (*P* = .483), consistent with the findings of Walker et al. [30]

Defecation function in RC is related to many factors, such as the degree of destruction of the pelvic floor muscle, level of anastomosis, degree of nerve damage in the pelvic floor, and radiotherapy and chemotherapy before and after surgery [31–33]. In our study, the defecation function outcomes of the 192 patients undergoing AR surgery, evaluated using the Wexner score, showed no significant difference between the two groups. Moreover, no significant differences were observed across the time periods (second year, third year, and fourth year). This may be attributed to excellent surgical skills, pelvic muscle training, and long-term recovery. The perception of colostomy-related problems and their impact on health-related QOL may differ between patients and healthcare professionals. Elfeki et al. [34] stated that the perspective of colostomy-related problems may differ between patients undergoing colostomy and healthcare professionals.

In our 192 AR surgery patients, we observed significant differences in RF, GQOL, and incidence of sleep disturbance and constipation between the non-ostomy and ostomy groups; the other indices in the functional and symptom areas showed no significant differences. The recovery of the RF, GQOL, and sleep disturbance and constipation indicators was slower than that of the other indicators, not only because of the surgical approach, comprehensive postoperative treatment, and hospital care, but also because of patients’ nutritional status, home care, habits, and psychological status. We used the EORTC QLQ-CR30 to analyze the quality of life of 31 patients with permanent ostomy across three time-periods, and found that the GQOL, dyspnea, and financial difficulty indicators were significantly different across the three time-periods. This is because the patients’ self-perception scores were significantly higher in the fourth year. We found that some patients had lung infections during this period; however, after the administration of anti-inflammatory agents, the use of dilated bronchial drugs could be alleviated, and there were few cases of lung metastasis. In our study, the highest number of cases of economic difficulties were observed in year 2 after surgery, as surgery and follow-up treatment, such as chemotherapy and radiotherapy, are expensive especiallyin the 31 permanent ostomy cases.

However, across the different time periods, the function score, including the PF, RF, EF, CF, SF, and GQOL, did not show significant differences between the non-ostomy and ostomy groups, although SF and GQOL showed significant differences in year 2. Therefore, we concluded that the PF, RF, EF, and CF of the ostomy patients were restored 1 years after surgery, while the SF and GQOL recovered more than 2 years after surgery. Our study found that most patients were highly optimistic and were spirited in their fight against the tumor, similar to the findings of Mareile [35] and others [36–40].

Brunet performed a similar study [41], which found no significant between-group differences in any of the symptom changes. Our study showed that symptom scores for fatigue, nausea and vomiting, pain, dyspnea, sleep disturbance, appetite loss, constipation, diarrhea, and financial difficulties in the ostomy group recovered in the second year after surgery. This may be related to the patients’ self-conditioning after surgery. In our follow-up, we found that many patients not only used allopathy but also used traditional Chinese medicine treatment after surgery. The symptom score curves of the two groups fluctuated in a narrow interval, with no statistically significant difference observed during the three different time spans (Fig. 4). Regarding preventive ostomy, both loop ileostomy (LI) and loop transverse colostomy have their own advantages and disadvantages. Owing to the lower wound infection rate, lower incidence of parastomal hernia, and shorter time to first defecation, LI is recommended for all patients except those with potential electrolyte disturbance and sensitive skin [42].

Our research has some limitations. The sample size was not large enough, and the study was not a prospective controlled randomized study. Considering that the interval time of closed operation after rectal surgery was within 1 year and because many previous studies have reported poorer defecation function and QOL in the short term, we did not analyze the defecation function and QOL within the 1 year following surgery and did not perform sequential follow-up. Although follow-up methods differed among participants (they were approached either by telephone or email or both), this did not affect outcomes. We did not include normal cases as a control group because such individuals were reluctant to participate in the study, and this was not a key aspect of our study.

## Conclusions

In summary, our study concluded that RC patients undergoing ostomy surgery, especially those with low and super-low RC, showed poorer defecation function and QOL. However, 2 years following surgery, most of the defecation function and QOL indicators were restored.

## Data Availability

Corresponding author can provide raw and analyzed data on request.

## Abbreviations

RC: Rectal cancer
QOL: Quality of life
AR: Anterior resection with anastomosis
TD: Tumor distance from the anal margin
AD: Anastomosis distance from the anal margin
CRP: C-reactive protein
SS: Standard score
PF: Physical function
RF: Role function
EF: Emotional function
CF: Cognitive function
SF: Social function
LI: Loop ileostomy
